# 600-ns pulsed electric fields affect inactivation and antibiotic susceptibilities of *Escherichia coli* and *Lactobacillus acidophilus*

**DOI:** 10.1186/s13568-020-00991-y

**Published:** 2020-03-18

**Authors:** Stacey L. Martens, Savannah Klein, Ronald A. Barnes, Patricia TrejoSanchez, Caleb C. Roth, Bennett L. Ibey

**Affiliations:** 1 Radio Frequency Bioeffects Branch, Bioeffects Division, Airman Systems Directorate, 711th Human Performance Wing, Air Force Research Laboratory, JBSA, Fort Sam Houston, San Antonio, TX USA; 2 Oak Ridge Institute for Science and Education, JBSA, Fort Sam Houston, San Antonio, TX USA; 3 Present Address: Department of Biological Sciences, University of South Carolina, Columbia, SC USA

**Keywords:** *E. coli*, *L. acidophilus*, Nanosecond pulsed electric fields, Antibiotic, Decontamination

## Abstract

Cell suspensions of *Escherichia coli* and *Lactobacillus acidophilus* were exposed to 600-ns pulsed electric fields (nsPEFs) at varying amplitudes (Low-13.5, Mid-18.5 or High-23.5 kV cm^−1^) and pulse numbers (0 (sham), 1, 5, 10, 100 or 1000) at a 1 hertz (Hz) repetition rate. The induced temperature rise generated at these exposure parameters, hereafter termed thermal gradient, was measured and applied independently to cell suspensions in order to differentiate inactivation triggered by electric field (E-field) from heating. Treated cell suspensions were plated and cellular inactivation was quantified by colony counts after a 24-hour (h) incubation period. Additionally, cells from both exposure conditions were incubated with various antibiotic-soaked discs to determine if nsPEF exposure would induce changes in antibiotic susceptibility. Results indicate that, for both species, the total delivered energy (amplitude, pulse number and pulse duration) determined the magnitude of cell inactivation. Specifically, for 18.5 and 23.5 kV cm^−1^ exposures, *L. acidophilus* was more sensitive to the inactivation effects of nsPEF than *E. coli*, however, for the 13.5 kV cm^−1^ exposures *E. coli* was more sensitive, suggesting that *L. acidophilus* may need to meet an E-field threshold before significant inactivation can occur. Results also indicate that antibiotic susceptibility was enhanced by multiple nsPEF exposures, as observed by increased zones of growth inhibition. Moreover, for both species, a temperature increase of ≤ 20 °C (89% of exposures) was not sufficient to significantly alter cell inactivation, whereas none of the thermal equivalent exposures were sufficient to change antibiotic susceptibility categories.

## Introduction

Bacterial contamination is recognized as a persistent and growing global concern for biotic and abiotic systems. To combat this threat, society has relied on three main bacterial inactivation methods: (1) Pharmaceutical (ex. antibiotics), (2) chemical (ex. antiseptics and disinfectants) and (3) physical (ex. heat, UV irradiation, cold plasma). Estimates from recent market research analyses indicate that these bacterial decontamination methods are so abundant, they globally comprise multibillion/million-dollar (US) industries at $46 billion (Watson 2019a), $5.5 billion (John 2017) and $149 million (Watson 2019b), respectively. Despite the large financial investments and modern technological advances bacteria continue to evolve and evade inactivation methods. Thus, novel bacterial control methods are urgently required.

One physical technique to inactivate microbes, which has been utilized for 100+ years (Sitzmann et al. 2017), is pulsed electric field(s) (PEF). Processes such as hospital effluent disinfection (Gusbeth et al. 2009), liquid and food pasteurization (Castro et al. 1993; Barbosa-Canovas et al. 2000), biofilm breakdown (Khan and El-Hag 2011; Freebairn et al. 2013) and topical burn antiseptic (Golberg et al. 2014) have found strong utilization for PEF. One of the main advantages for this inactivation process, versus the other available techniques, is the lack of resistance to the inactivation mode of action—membrane charging with subsequent, permanent breakdown of the cell wall (Katsuki et al. 2002; Dermol and Miklavčič 2017), also known as irreversible electroporation. However, the main disadvantage of this technology has been the large thermal gradient that is generated by the long (µs to > 1 s) pulses utilized, drastically restricting the use of this technology to heat-tolerant products. However, newly engineered nanosecond pulse generators, which create a significantly reduced thermal gradient, are now commercially available and have the potential to expand the PEF technology as a bacterial decontamination technique for heat-sensitive applications and products. This type of technology may prove especially useful for in vivo medical applications in which the subsequent heating of the surrounding tissue would be deleterious, as well as industrial food processing applications which require lower heat thresholds to maintain the sensory and physical properties of the food product (Barbosa-Canovas et al. 2000).

In this research study we examined the effects of a sub-microsecond pulse—600 ns, on two ubiquitous bacterial species, *Escherichia coli* and *Lactobacillus acidophilus*. We evaluated the impact of increasing the applied amplitude and pulse number to establish a range of inactivation thresholds at various exposure parameters. Given the effectiveness of nsPEF to inactivate bacteria, we hypothesized that the electroporated bacterial cells would be more sensitive to various antibiotics. To test this hypothesis, we exposed the treated cells to antibiotic-soaked discs and measured subsequent zones of growth inhibition. Furthermore, we measured the thermal gradient produced by each exposure and independently applied that thermal equivalent (TE) to both the inactivation and antibiotic susceptibility experiments. These results would validate that the observed inactivation and increase in antibiotic susceptibility was not based on thermal loading of the samples. We postulate that as a non-pharmaceutical, non-chemical, non-ionizing, and E-field driven technology, nsPEF has the potential to singularly or synergistically affect antibiotic treatment therapies, most especially antibiotic-resistant superbugs.

## Materials and methods

### Cell lines

*Escherichia coli* (ATCC 11775-MINI-PACK) and *Lactobacillus acidophilus* (ATCC 4357) cultures were purchased from American Type Culture Collection (ATCC) and propagated according to manufacturer’s protocol. Stocks were preserved by aliquoting respective bacterial broth with 50% glycerol (1:1) into 1.5 mL tubes and kept frozen at − 80 °C.

### Exposure system

**Fig. 1:**
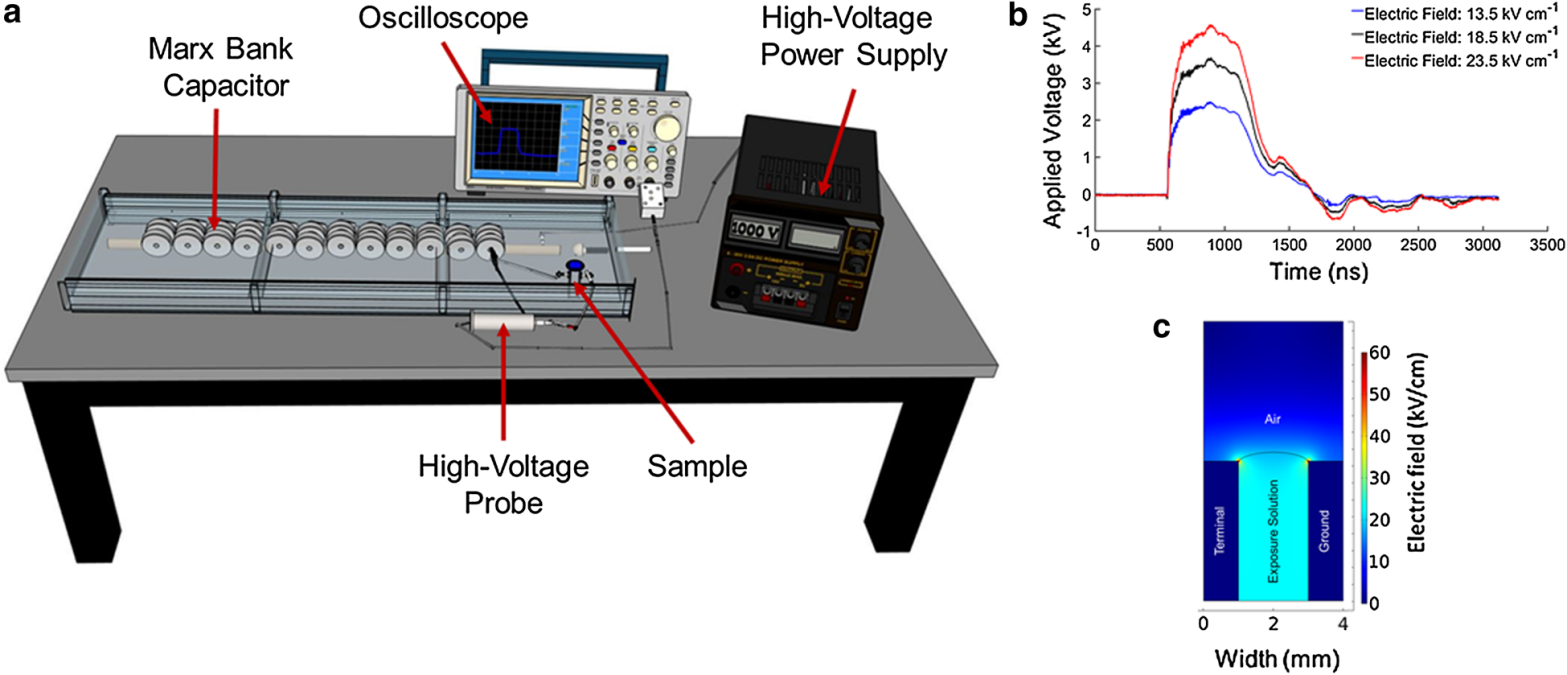
nsPEF exposure characterization. A schematic of the Marx bank capacitor system used to deliver the 600-ns PEF is provided (**a**). Representative oscilloscope traces of the applied voltages used to deliver an E-field amplitude of 13.5 (blue), 18.5 (black) or 23.5 (red) kV cm^−1^ (**b**) are shown. COMSOL Multiphysics^®^ software was used to model the predicted E-field throughout the electroporation cuvette (both the x and y axis are equal in scale) (**c**)

A Marx bank capacitor system (Fig. 1a), previously described (Ibey et al. 2014; Cantu et al. 2016), was used to generate the 600-ns unipolar pulse (Fig. 1b). A high-voltage probe (#P6015A Tektronix, Beaverton, OR, USA) connected to a high-speed oscilloscope (#TDS 30504B Tektronix, Beaverton, OR, USA) was used to measure the delivered pulse. For all exposures except 1000 pulses, the applied voltage from the high-voltage power supply was set at a fixed voltage to achieve the desired mean E-field amplitude in the cuvette (13.5 ± 0.6, 18.5 ± 2.5 and 23.5 ± 2.3 kV cm^−1^). For the 1000 pulse exposures, a rise in the temperature decreased the impedance of the cuvette resulting in a lower applied field. To maintain the mean exposure amplitude, the applied voltage was manually adjusted periodically. While this adjustment is not ideal, it ensured a more uniform E-field exposure, but likely contributed to some of the sample variability observed for 1000 pulse exposures. COMSOL Multiphysics^®^ software v. 4.3b (COMSOL) was used to model E-field distribution within the exposure cuvette. The predicted E-field distribution displays uniformity throughout the exposure solution (Fig. 1c) thus the E-field is assumed to be spatially uniform throughout cellular exposures.

### Cell culture

Growth media was purchased from Becton, Dickson and Company (BD) (Franklin Lakes, NJ, USA). Nutrient broth (NB) (BD #213000) or De Man, Rogosa and Sharpe (MRS) broth (BD #288210) were utilized to culture *E. coli* and *L. acidophilus*, respectively. Overnight cultures were initiated by inoculating one vial from the − 80 °C stock into 50 mL of NB or MRS and incubated at 37 °C overnight. *E. coli* was cultured aerobically at 250 rpm. *L. acidophilus* was cultured in a capnophilic environment with elevated carbon dioxide levels using a BD GasPak 150 system (BD #260629), stationary. The following morning the optical density of a 100 µL aliquot was read in a spectrophotometer (#16032324 Synergy HTX BioTek, Winooski, VT, USA) set to a wavelength of 600 nm. To initiate experiments, a fresh 50 mL of growth media was inoculated with enough overnight culture to provide a starting optical density value of 0.05. Cultures were returned to their respective incubators and allowed to grow to log phase (approx. 4 h).

### Exposures

To ensure consistency in exposure conditions both species were pelleted, washed and re-suspended in a 0.85% sodium chloride (NaCl) exposure solution (pH = 6.0, Conductivity = 1.33 S m^−1^). Final cell concentration was adjusted to approx. 1.5 × 10^8^ cells mL^−1^. All cell solutions started at room temperature (approx. 22 °C).

(i) nsPEF induced temperature change (ΔT) (Thermal Gradient): A 400 µL sample of 0.85% NaCl solution was pipetted into a 2 mm aluminum, parallel plate electroporation cuvette (#89047-208 VWR International, Radnor, PA, USA). Prior to initiating the nsPEF exposure, a resting temperature (°C) of the NaCl solution was obtained by inserting a K-type thermocouple probe (#N1 USB-TC01 National Instruments, Austin, TX, USA) into the solution and then recorded using the N1 temperature logger software V1.1 (National Instruments, Austin, TX, USA). After the measurement, the probe was removed and the NaCl solution was exposed to a random series of: 1, 5, 10, 100 or 1000 pulses at a 1 Hz (one pulse/second) repetition rate and the amplitude was varied to 13.5, 18.5 or 23.5 kV cm^−1^. Sham exposures were performed by loading the cuvettes with NaCl solution and placing it into the pulser for 8 min (median time of the longest exposure) but no pulse was delivered. Immediately following exposure, the probe was re-inserted into the NaCl solution and the post exposure temperature was recorded. Change in temperature (ΔT) was obtained by subtracting the pre-exposure temperature from the post exposure temperature.
(ii) nsPEF: A 400 µL cell suspension (cells plus 0.85% NaCl) was pipetted into a 2 mm aluminum, parallel plate electroporation cuvette (#89047-208 VWR International, Radnor, PA, USA) and exposed to the above-mentioned exposure parameters.
(iii) nsPEF-induced thermal equivalent (TE) (heat stress): In total, three cell suspensions (cells suspended in 0.85% NaCl) of 120 µL each were pipetted into 3–200 µL PCR tubes (#TCS0803 BioRad, Hercules, CA, USA), capped (#TLS0801 BioRad, Hercules, CA, USA) and placed into an Eppendorf Mastercycler gradient thermocycler (#5331 Eppendorf, Hamburg, Germany). A resting temperature (°C) of the cell solution was obtained by inserting the thermocouple into a fourth PCR tube containing only 0.85% NaCl solution and recorded as mentioned above. The thermocycler was then set to the appropriate ΔT as observed in the thermal gradient experiment. Thermal exposure time was equivalent to nsPEF exposures (1 Hz) and was initiated once the ultimate temperature was reached in the thermocycler.

### Enumeration of colony forming units (CFU)

Following the nsPEF or TE exposures, cells were removed from their respective exposure container and placed into a 1.5 mL tube. A tenfold serial dilution was performed by inoculating 900 µL of distilled water with a 100 µL aliquot from the previous dilution (no dilution to 10^6^). Then, a 100 µL aliquot of the 10^4^ to 10^6^ samples were spread onto 20 mL of the respective growth agar using the spread plate technique. Following a 24 h incubation period, a direct colony count was taken. Log reduction of CFU was calculated by taking log_10_(Sham/Exposed). Lethal concentration (LC_50_) values were calculated using probit analysis in Excel; mean% dead values (across n experiments) were utilized to determine probit values.

### Antibiotic susceptibility

A 100 µL aliquot of cell solution was removed from the no dilution Eppendorf tube (see above) and spread using the spread plate technique. The following antibiotic-soaked discs, purchased from BD, were subsequently stamped onto the inoculated *E. coli* and *L. acidophilus* plates: kanamycin (30 µg) (BD #B31301), tobramycin (10 µg) (BD #B31569), vancomycin (30 µg) (BD #B31353), ampicillin/sulbactam (10/10 µg) (BD #B31660), aztreonam (30 µg) (BD #B31641) and tetracycline (30 µg) (BD #B31344). The diameter of the zones of growth inhibition were measured (mm) following a 24 h incubation period and the bacteria were classified as:

- Resistant (R): “Clinical efficacy has not been reliably shown in treatment studies” (Hardy Diagnostics 1996)
- Intermediate (I): “Clinical applicability in body sites where the drug is physiologically concentrated or when a higher than normal dosage of the drug can be used. The MIC of the isolate may approach usually attainable blood and tissue levels but the response rate may be lower than for susceptible isolates” (Hardy Diagnostics 1996) or
- Susceptible (S): “An infection due to the organism may be treated with the concentration of antimicrobial agent used, unless otherwise contraindicated” (Hardy Diagnostics 1996)

based on the Clinical and Laboratory Standards Institute (CLSI) pre-existing guidelines which were previously described by Hardy Diagnostics (1996) and Charteris et al. (1998).

### Statistical analysis

*Escherichia coli* nsPEF experiments were performed 6 times (n = 6). All remaining experimental conditions (thermal gradient, nsPEF, TE, antibiotic susceptibility) were performed in three independent replicates (n = 3). Unless otherwise indicated, values represent the mean of n plus the standard error (SE). A student’s T-test with a p-value ≤ 0.05 was carried out for all experiments. A one-tail test was utilized for the thermal gradient experiments while a two-tail test was utilized for the nsPEF and TE experiments. A statistically significant difference is represented by an asterisk.

## Results

### Amplitude and pulse number affect 600-ns PEF thermal gradient

**Fig. 2:**
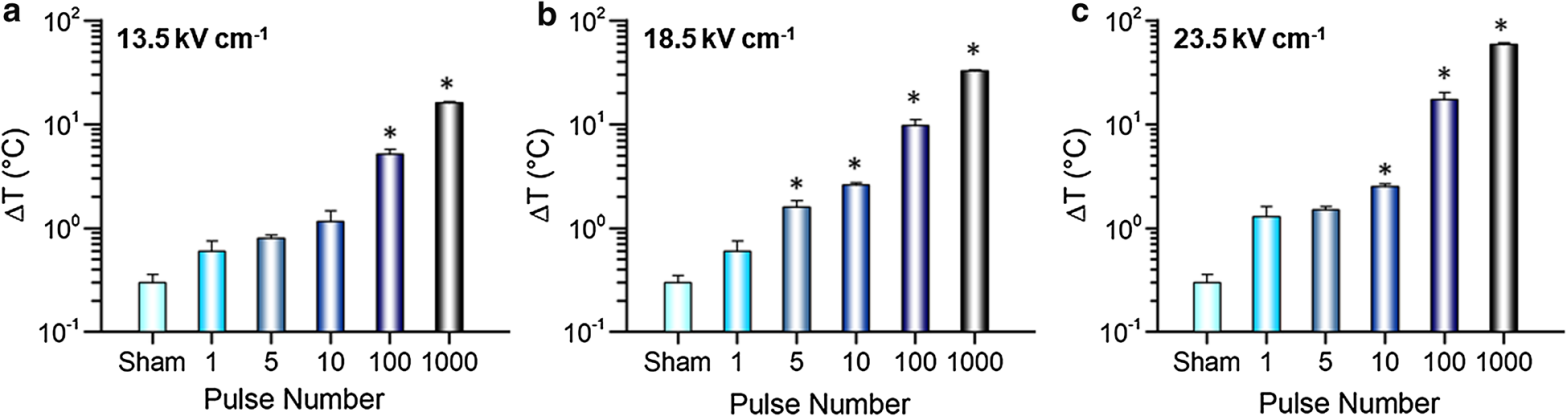
nsPEF induced temperature change. Temperature change (ΔT) (°C) of 0.85% NaCl cell solution exposed to a 600-ns PEF at 13.5 (**a**), 18.5 (**b**) or 23.5 (**c**) kV cm^−1^ at 0 (sham), 1, 5, 10, 100 or 1000 pulses. Results are the mean ± SE of three independent experiments (n = 3). An asterisk represents a statistically significant difference from the sham

The temperature of the cell exposure solution (0.85% NaCl) was measured for each amplitude and pulse number used in this study (Fig. 2a–c). As expected, increasing the amplitude or pulse number resulted in an increase in the overall thermal gradient delivered to the sample. However, only two conditions: 1000 pulses, at 18.5 and 23.5 kV cm^−1^ produced a large enough ΔT capable of initiating *irreversible* bacterial inactivation (ΔT: 33 and 59 °C, respectively) (World Health Organization 2018; Bull et al. 2013).

### nsPEF inactivate *E. coli* and *L. acidophilus* to a greater extent than their thermal equivalents (TE)

The inactivation of *E. coli* and *L. acidophilus*, as measured by CFU, was evaluated after a 600-ns PEF or its TE at various amplitudes and pulse numbers.

**Fig. 3:**
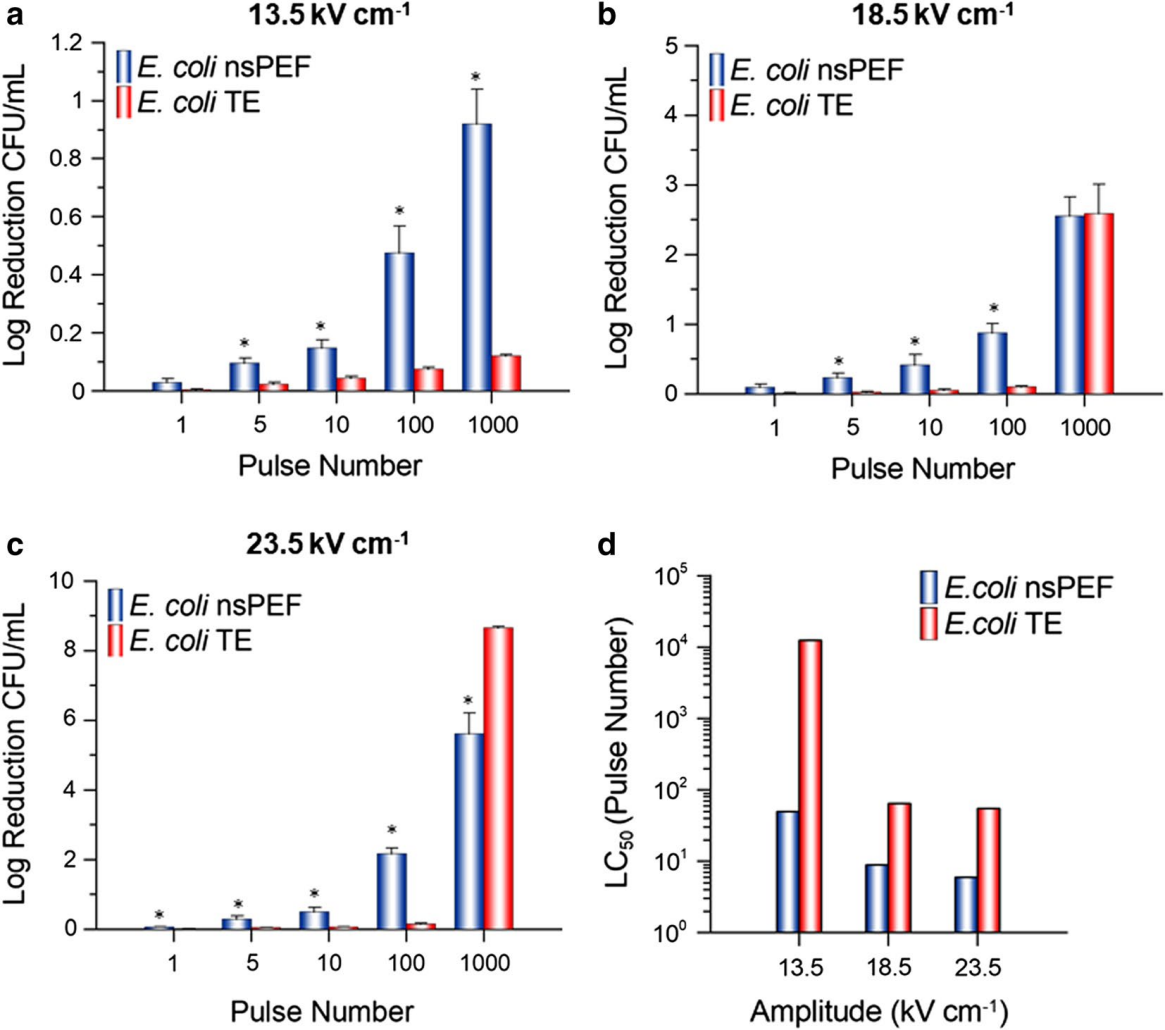
*E. coli* cell inactivation. Cell inactivation (log reduction CFU mL^−1^) (**a**–**c**) and LC_50_ values (**d**) for *E. coli* exposed to a 600-ns PEF (blue) or its respective TE (red) at an amplitude of 13.5, 18.5 or 23.5 kV cm^−1^ and 1, 5, 10, 100 or 1000 pulses. nsPEF results are normalized to the sham and are the mean plus SE of six independent experiments (n = 6). TE results are normalized to the sham and are the mean plus SE of three independent experiments (n = 3). LC_50_ values were calculated by utilizing the mean log reduction CFU mL^−1^ at each amplitude. An asterisk represents a statistically significant difference (p ≤ 0.05) between the nsPEF and TE exposure for that pulse number

**Fig. 4:**
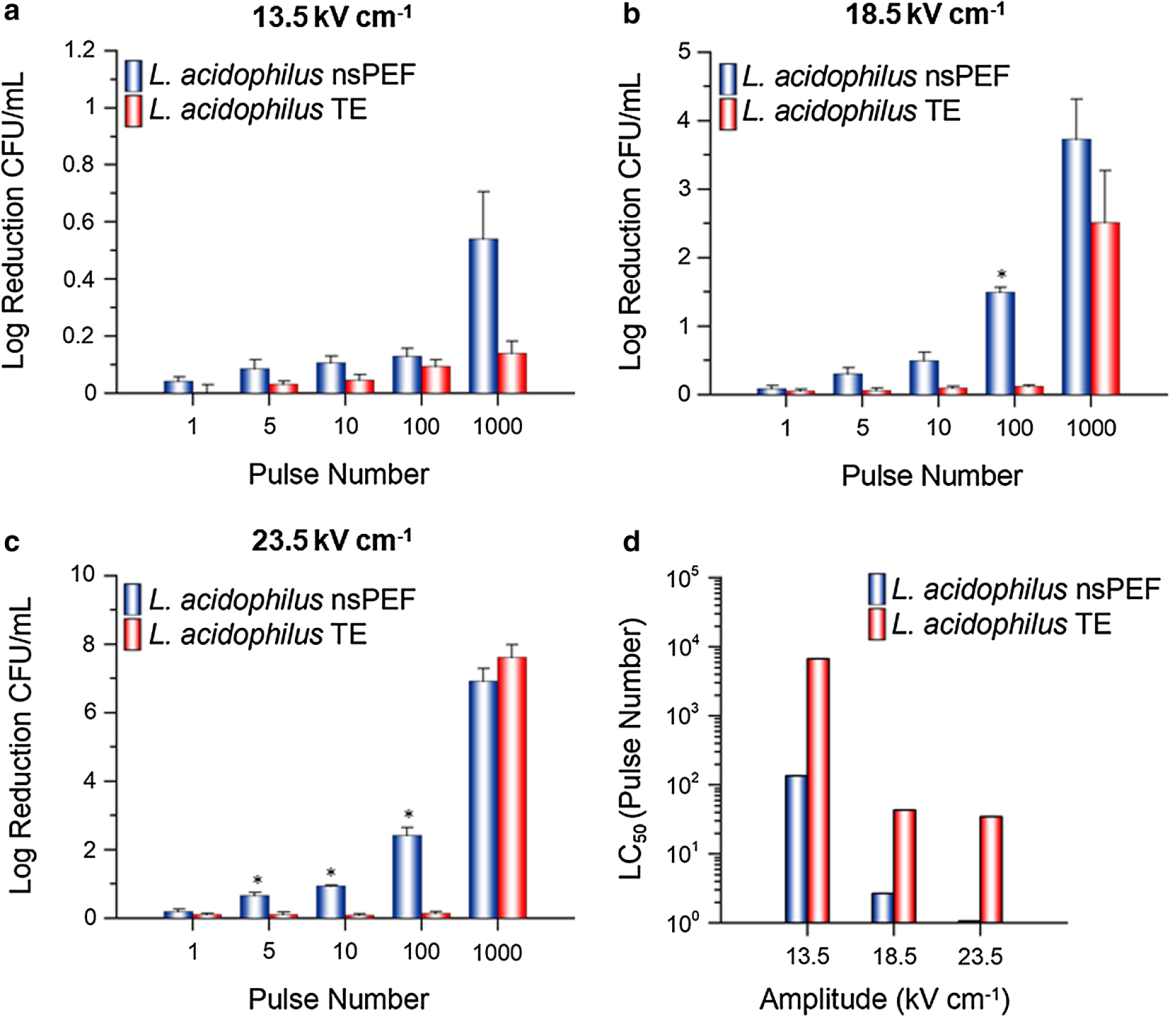
*L. acidophilus* cell inactivation. Cell inactivation (log reduction CFU mL^−1^) (**a**–**c**) and LC_50_ values (**d**) for *L. acidophilus* exposed to a 600-ns PEF (blue) or its respective TE (red) at an amplitude of 13.5, 18.5 or 23.5 kV cm^−1^ and 1, 5, 10, 100 or 1000 pulses. nsPEF and TE results are normalized to the sham and are the mean plus SE of three independent experiments (n = 3). LC_50_ values were calculated by utilizing the mean log reduction CFU mL^−1^ at each amplitude. An asterisk represents a statistically significant difference (p ≤ 0.05) between the nsPEF and TE exposure for that pulse number
(ii) TE exposures: To better understand if the observed increase in cell inactivation was due in-part to the nsPEF-induced thermal gradient, both species were subjected to a TE from each amplitude and pulse number examined (Figs. 3a–c and 4a–c). Only two exposure parameters: 1000 pulses at 18.5 and 23.5 kV cm^−1^ produced a substantial log reduction of CFU for *E. coli* (Fig. 3b, c, red) and *L. acidophilus* (Fig. 4b, c, red). However, this was expected as the thermal gradient experiment resulted in a change in temperature above the normal physiological range for both species. For the remainder of the exposures (88% of total exposures examined), log reduction CFU remained below 0.15, meaning ≥ 87% viability.

(i) nsPEF exposures: For both *E. coli* (Fig. 3a–c, blue) and *L. acidophilus* (Fig. 4a–c, blue), inactivation was positively correlated to amplitude and/or pulse number. In general, cell inactivation increased as amplitude and/or pulse number increased. A maximum log reduction of CFU mL^−1^ was achieved at 1000 pulses for all amplitudes examined (a. 13.5 kV cm^−1^: 0.9, 0.5) (b. 18.5 kV cm^−1^: 2.5, 3.7) (c. 23.5 kV cm^−1^: 5.6, 6.9) for *E. coli* and *L. acidophilus*, respectively.

### nsPEF increase the efficacy of various antibiotics on *E. coli* and *L. acidophilus*

The effectiveness of various antibiotics was examined for nsPEF and the TE exposures by measuring zones of inhibition following a 24 h incubation. Table 1 provides results for which the antibiotic susceptibility of the sham began as resistant (R) or intermediate (I), and the exposed sample transitioned to intermediate (I) or susceptible (S).

**Table 1:** Zones of growth inhibition for *E. coli* and *L. acidophilus*

Pulse #	*E. coli*								*L. acidophilus*							
	Kanamycin				Tobramycin				Vancomycin				Ampicillin–sulbactam			
	nsPEF	SD	TE	SD	nsPEF	SD	TE	SD	nsPEF	SD	TE	SD	nsPEF	SD	TE	SD
*13.5 kv cm*^*−1*^																
Sham	16 (I)	0.2	16 (I)	0.2	11 (R)	0.6	11 (R)	0.6	13 (R)	0.6	13 (R)	0.6	14 (I)	0.6	14 (I)	0.6
1	16 (I)	0	16 (I)	0	11 (R)	0	11 (R)	0.6	13 (R)	0	14 (R)	0	14 (I)	1	14 (I)	0
5	16 (I)	0	16 (I)	0	11 (R)	0	11 (R)	0.6	13 (R)	1	14 (R)	0.6	15 (I)	1	14 (I)	0.6
10	16 (I)	0	16 (I)	0	11 (R)	0.6	11 (R)	0.5	13 (R)	1	14 (R)	0.6	15 (I)	1	14 (I)	1
100	16 (I)	0	16 (I)	0	12 (R)	0	12 (R)	0.6	14 (R)	1	14 (R)	0	16 (S)	0.7	14 (I)	0
1000	18 (S)	0.3	16 (I)	0	12 (R)	0	11 (R)	0.6	16 (I)	1	14 (R)	0.3	19 (S)	1	14 (I)	0.6
*18.5 kv cm*^*−1*^																
Sham	16 (I)	0.2	16 (I)	0.2	11 (R)	0.6	11 (R)	0.6	13 (R)	0.6	13 (R)	0.6	14 (I)	0.6	14 (I)	0.6
1	17 (I)	1	16 (I)	0.3	11 (R)	0.6	12 (R)	0.3	13 (R)	0.6	14 (R)	0	14 (I)	0	14 (I)	0.6
5	18 (S)	0	16 (I)	0.6	12 (R)	0	12 (R)	0.6	13 (R)	0.6	14 (R)	0	14 (I)	1	14 (I)	0.3
10	19 (S)	0.6	16 (I)	0	12 (R)	0	12 (R)	0	13 (R)	0	14 (R)	1	14 (I)	1	14 (I)	0
100	20 (S)	0	16 (I)	0.6	12 (R)	0.6	12 (R)	0.6	14 (R)	1	14 (R)	0.8	16 (S)	0	14 (I)	1
1000	22 (S)	0	16 (I)	0	14 (I)	0.6	11 (R)	0	15 (I)	0	14 (R)	0	19 (S)	1.4	14 (I)	0
*23.5 kv cm*^*−1*^																
Sham	16 (I)	0.2	16 (I)	0.2	11 (R)	0.6	11 (R)	0.6	13 (R)	0.6	13 (R)	0.6	14 (I)	0.6	14 (I)	0.6
1	17 (I)	0.6	16 (I)	0	11 (R)	0	11 (R)	0.6	14 (R)	0	14 (R)	0.3	14 (I)	2	14 (I)	0
5	17 (I)	0	16 (I)	0.6	11 (R)	1.2	11 (R)	0.6	15 (I)	0.6	14 (R)	0.6	15 (I)	0.8	14 (I)	0.3
10	19 (S)	1	16 (I)	0	12 (R)	1	11 (R)	0.6	15 (I)	0.6	14 (R)	0.3	16 (S)	0.6	14 (I)	0.3
100	22 (S)	0.5	16 (I)	0	16 (S)	0	12 (R)	0.5	15 (I)	1	14 (R)	0.6	18 (S)	1	14 (I)	0
1000	*	*	*	*	*	*	*	*	*	*	*	*	*	*	*	*

Following a 13.5, 18.5 or 23.5 kV cm^−1^ 600-ns PEF and its respective TE, *E. coli* and *L. acidophilus* were exposed to the following antibiotic-soaked discs: kanamycin (30 µg) and tobramycin (10 µg) and vancomycin (30 µg) and ampicillin/sulbactam (10/10 µg), respectively. Measured zones of growth inhibition (diameter, mm) are reported. Values represent the mean of 3 replicates, the standard deviation (SD) of each value is provided. Susceptibility is expressed as R (resistant), I (intermediate) or S (susceptible). No observable growth is indicated by an asterisk (*)

(i) *E. coli*: Zones of inhibition for kanamycin (30 µg) or tobramycin (10 µg) were larger when nsPEF was applied prior to the treatment. The kanamycin treated samples transitioned from intermediate to susceptible as early as 10 pulses in the 18.5 and 23.5 kV cm^−1^ exposures. “Susceptible” was also attained in the 13.5 kV cm^−1^ amplitude however, it was not achieved until 1000 pulses. The tobramycin treated samples transitioned from resistant to susceptible by 100 pulses in the 23.5 kV cm^−1^ exposure. Additionally, a transition from resistant to intermediate was observed in the 1000 pulse 18.5 kV cm^−1^ sample.
(ii) *L. acidophilus*: Zones of inhibition for vancomycin (30 µg) or ampicillin-sulbactam (10/10 µg) were larger when nsPEF was applied prior to the treatment. The vancomycin treated samples transitioned from resistant to intermediate as early as 5 pulses in the 23.5 kV cm^−1^ exposure. Intermediate susceptibility was also observed by the two lower amplitudes examined; however, it was not achieved until 1000 pulses. The ampicillin-sulbactam treated samples transitioned from intermediate to susceptible as early as 10 pulses in the 23.5 kV cm^−1^ exposure. “Susceptible” was also attained in the other two amplitudes examined however it was not achieved until 100 pulses.

For both species, a measurement for the 1000 pulse 23.5 kV cm^−1^ exposures could not be verified as there was no bacterial growth on the culture plate, indicative of total cell inactivation by either nsPEF, the thermal gradient or a combination of the two. Moreover, for all three amplitudes and four antibiotics reported in Table 1, none of the TE exposures were sufficient to change the susceptibility indicating that under these parameters’ inactivation was directly related to E-field effects and not a thermal gradient.

## Discussion

In the present study we show in vitro results for the efficacy of 600-ns PEF to inactivate *Escherichia coli* and *Lactobacillus acidophilus*. While the effects of nsPEF have been studied on *E. coli* (Chalise et al. 2006; Perni et al. 2007; Guionet et al. 2014, 2015; Novickij et al. 2018) and other species such as *Staphylococcus aureus* (Chaturongakul and Kirawanich 2012; Vadlamani et al. 2018; Novickij et al. 2019), *Salmonella typhimurium* (Perni et al. 2007) and *Bacillus subtilis* (Katsuki et al. 2002); we chose to compare the effects of *E. coli* with *L. acidophilus* for two reasons. First, several studies have suggested that cell size and shape play a critical role in transmembrane potential charging and subsequent membrane pore formation (Kandušer and Miklavčič 2008; Khan and El-Hag 2011). Thus, to reduce effects resulting from differences in size and shape we chose two morphologically similar species; both species are rod shaped with similar size dimensions (Hardy Diagnostics 1996–2016; Reshes et al. 2008). Second, as we wanted to compare the effects of two physiologically different species (Gram negative and Gram positive), we chose the Gram-positive *L. acidophilus*, as it is generally considered to be apathogenic and moreover a probiotic. Furthermore, to our knowledge, we are the first group to report nsPEF inactivation results for *L. acidophilus*. Comparing the species examined here is important for potential applications where nsPEF exposures could be utilized to selectively target a bacterial species. For example, in vivo medical applications where a mixed population of pathogens and non-pathogens exist or in situ for food sterilization/liquid pasteurization purposes.

For comparison of cell inactivation thresholds between *E. coli* and *L. acidophilus* we utilized a range of exposure parameters including: E-field amplitude (Low-13.5, Mid-18.5 and High-23.5 kV cm^−1^) and pulse number (0 (sham), 1, 5, 10, 100 or 1000 pulses). This range of exposure conditions allowed us to determine parameter combinations that could be used to selectively target each species respectively. Furthermore, as studies have suggested that parameters of the exposure solution (ex. pH, conductivity) are equally important as cell type to electropermeabilization metrics (Kotnik et al. 1997; Stoodley et al. 1997), we chose to remove the cells from their respective growth media and re-suspend each cell type into a saline exposure solution; thus limiting potential differences based on differences in media composition. Our results show that, in general, *L. acidophilus* was more susceptible to the 600-ns PEF than *E. coli*; mean nsPEF LC_50_ values were higher for *E. coli* (Fig. 3d, blue) than *L. acidophilus* (Fig. 4d, blue) for both the 18.5 and 23.5 kV cm^−1^ exposures. However, this was not the case for the 13.5 kV cm^−1^ nsPEF exposures, suggesting that there may be an E-field threshold for inactivation of *L. acidophilus*.

These results were intriguing to us as many historical studies have suggested that based on differences in the electrical properties of the peptidoglycan layer for Gram-negative and Gram-positive species, a Gram-negative bacterium should be more susceptible to the effects of PEF (Hülsheger et al. 1983). However, a review of more recent PEF studies has proposed that this may not always be the case. For instance, García et al. (2005) examined the viability of various bacterial species to PEF treatments in an exposure medium with a pH of 4 or 7. Their results suggested that the same cell type will display inactivation differentially based on the pH of the exposure media (García et al. 2005). While the emphasis of this manuscript was on the effects of the E-field and temperature, we also examined the effect that the 600-ns PEF would have on the pH of the unbuffered NaCl exposure media. Utilizing the largest amplitude of 23.5 kV cm^−1^ we measured the *final* pH from all pulse numbers utilized, as this would display the most extreme pH changes. We observed the pH increase as the pulse number increased: 6.0 (sham), 6.3 (1 pulse), 6.5 (5 pulses), 6.8 (10 pulses), 7.9 (100 pulses) and 8.9 (1000 pulses). It is important to note that these pH changes are attributed to electrochemistry within the cuvette related to the aluminum electrodes and therefore would scale with amplitude. Therefore, the pH measurements included in this paper represent the worst-case scenario for the bacteria and would be significantly less for the other two lower amplitudes. However, for exposures at an amplitude of 23.5 kV cm^−1^ and pulses ≤ 10, the cumulative change in pH is rather modest, therefore the observed impact on cells would likely be independent of pH. For the 100 and 1000 pulse exposures, the pH change is significant. Although, it is important to note that this pH is achieved over the course of the pulse application and the bacteria only experience the extreme pH for a short duration between end of exposure and analysis. This is in contrast to García et al. (2005), where the bacteria are placed into a buffer already adjusted to a set pH level. Furthermore, for 1000 pulse exposures, where the pH shows its largest increase, temperature also increases making isolation of nsPEF-specific effects on the cells difficult. This is a topic that we would like to further investigate in future studies.

Furthermore, as it is theorized that inactivation is directly related to pore formation in the membrane (Tieleman et al. 2003; Thompson et al. 2016; Meglic and Kotnik 2016), our results correlate well to a study conducted by Piggot et al. (2011) in which molecular dynamic simulations of electroporation of the Gram-negative *E. coli* outer membrane and the Gram-positive *S. aureus* membrane were conducted (Piggot et al. 2011). In these simulations, the *E. coli* outer membrane was more resistant to poration than the *S. aureus* membrane; the higher resistance was attributed to reduced mobility of the lipopolysaccharide molecules such that the phospholipids were required to fill the water-filled pore resulting in lipid flip-flop (Piggot et al. 2011). The results from that simulation study suggest that other Gram-positive species, such as *L. acidophilus*, may be susceptible to electroporation and subsequent inactivation more easily than *E. coli*. Nevertheless, our results indicate that under these exposure conditions it was ultimately the total energy delivered (amplitude, pulse number and pulse duration) that determined the extent of cell inactivation for an individual species.

Although nsPEF technology is largely considered to be E-field driven, during treatment there is an energy-dependent increase in process temperature due to electric current flow and individual product resistance (Schottroff et al. 2018); thus, additional inactivation effects due to heating should be considered. Consequently, for temperature-sensitive applications, it was important to differentiate inactivation triggered by the E-field from the thermal gradient. Additionally, beneficial compounds (e.g. antimicrobial agents, nutrients, etc.) found within target materials and their physical properties may also be affected by temperature gradients induced by extreme PEF exposures. Although the greatest extent of cell inactivation was achieved by 1000 pulses at 18.5 and 23.5 kV cm^−1^; for temperature-sensitive applications, we postulate that the more suitable exposures would be ≤ 100 pulses. At these pulse numbers a significant amount of cell inactivation was obtained by the nsPEF exposure while the thermal gradient and subsequent TE inactivation was negligible. Furthermore, the 13.5 kV cm^−1^ amplitude produced a greater inactivation difference between *E. coli* and *L. acidophilus*, while greatly restricting the thermal gradient, at 100 and 1000 pulses. These parameters might be especially useful to target one cell type over the other. However, an important item to note from this study is that the results from the TE exposures reflect a more severe result than what the nsPEF exposed cells actually experienced. This difference can mainly be attributed to the manner by which the nsPEF or TE cells were delivered heat. For instance, during the nsPEF exposures a 1 Hz repetition rate was directly correlated to exposure time (i.e. it took 5 s to deliver 5 pulses). However, for the TE exposures, there was a rate differential by which the thermocycler would adjust temperature gradients between “high” and “low” temperatures. For example, exposures which were set to a higher temperature (ex. 100 or 1000 pulses) were heated more quickly than samples that were set to a lower temperature (ex. 1, 5, or 10 pulses). Therefore, to maintain consistency throughout the experiment, the exposure time (equivalent to 1 Hz) was only initiated after a final temperature was achieved. This resulted in a longer total exposure time for the TE exposures as compared to the nsPEF exposures. Interestingly, although the TE LC_50_ values show slight variation at the different amplitudes between *E. coli* (Fig. 3d, red) and *L. acidophilus* (Fig. 4d, red), a similar trend can be observed in which the low amplitude exposures (13.5 kV cm^−1^) require more pulses to kill 50% of the population. However, once a heat threshold was met by pulse number the applied amplitude (18.5 vs. 23.5 kV cm^−1^) became irrelevant.

Lastly, as there is an urgent need for novel techniques to combat antibiotic resistant microbes (CDC 2019), we investigated the possible synergistic effects that nsPEF or the TE exposures could have on antibiotic susceptibilities. To examine this, we exposed both the nsPEF and TE exposed cells to four classes (aminoglycoside, tetracycline, glycopeptide and β-lactams) of antibiotic soaked disks and allowed the bacteria to grow overnight as the antibiotic diffused throughout the media. Results in Table 1 show that multiple nsPEF exposures can enhance the susceptibility of antibiotics for both *E. coli* and *L. acidophilus*, indicative by increased zones of growth inhibition. We also display results for antibiotics that, for *E. coli*, originated as susceptible but displayed increased zones of inhibition for select nsPEF exposures (Additional file 1: Table S1). Although there were some minor increases in the zones of inhibition for the TE exposures, none of these treatments were significant enough to provide a change in susceptibility, indicating that the change in antibiotic susceptibility was due to the effects of the E-field and not to any induced heating. Although the different classes of antibiotics work on various cellular targets to inactivate bacteria (Li et al. 2015; Kapoor et al. 2017), the first step in the mechanism of action for all antibiotics is to traverse the cell membrane and enter the cytoplasm (James et al. 2009; Krause et al. 2016). Research conducted by Pillet et al. (2016) showed scanning electron, transmission electron and atomic force microscopy results demonstrating the morphological, mechanical and physical damage to the cell wall of *Bacillus pumilus* from various strengths of µs electric pulses (Pillet et al. 2016). These results strongly support the theory that PEF directly impact bacterial cell wall integrity. Therefore, we would conclude that damage to the integrity of the cell wall and nsPEF-induced poration of the membrane is reducing or removing this first step allowing for the translocation of the antibiotic to be easier, faster and thus more effective. However, these results were limited to testing the bacterial susceptibility to various antibiotics only after the nsPEF exposure was delivered. It is likely that the addition of antibiotics to the pulsing medium could have increased the efficiency of cell death, but it would have also introduced the possibility of direct electrotransfer of the antibiotic into the cell, which was intentionally avoided in this study. Future research should evaluate if electrotransfer of antibiotics during nsPEF exposure increases the level of death across the various bacterial species.

In conclusion, this study has shown the efficacy of 600-ns PEF, at various amplitudes and pulse numbers, to inactivate cells and enhance the susceptibility of *E. coli* and *L. acidophilus* to various antibiotics. These results have aided in further building a foundation for the utilization of nsPEF in heat-sensitive bacterial inactivation applications and as a possible tool to combat antibiotic resistant microbes. Based on the positive results of this study, future investigations into the utilization of nsPEF for decontamination with medically relevant anaerobic species like *Clostridium difficile* or species with special pathogenic traits like the capsular polysaccharide matrix of *Klebsiella* should also be investigated. Finally, although there does not currently seem to be resistance mechanisms by the bacteria to evade PEF inactivation, there is limited research on the topic. Future studies should include an exhaustive attempt to further understand bacterial resistance to PEF.

## Supplementary information

**Additional file 1: Table S1.** Zones of growth inhibition for *E. coli*.

## Data Availability

Corresponding author could provide all the experimental data on valid request.

## Abbreviations

ns: Nanosecond
nsPEF: Nanosecond pulsed electric field(s)
kV: Kilovolts
cm: Centimeter
Hz: Hertz
E-field: Electric field
h: Hour
PEF: Pulsed electric field(s)
µs: Microsecond
s: Second
TE: Thermal equivalent
mL: Milliliter
C: Centigrade
BD: Becton, Dickson and Company
NB: Nutrient broth
MRS: De Man, Rogosa and Sharpe
rpm: Rotations per minute
µL: Microliter
nm: Nanometer(s)
approx: Approximately
NaCl: Sodium chloride
pH: Potential hydrogen
S/m: Siemens per meter
Δ: Delta or change in
mm: Millimeter
PCR: Polymerase chain reaction
CFU: Colony forming unit(s)
LC_50_: Lethal concentration 50%
µg: Micrograms
R: Resistant
I: Intermediate
S: Susceptible
SE: Standard error
